# The influence of human agency beliefs on ascribing gaze-signalled communicative intent

**DOI:** 10.1038/s41598-025-22810-9

**Published:** 2025-10-17

**Authors:** Friederike Charlotte Hechler, Emmanuele Tidoni, Emily S. Cross, Nathan Caruana

**Affiliations:** 1 Macquarie University, Balaclava Rd, Macquarie Park, NSW 2109 Australia; 2 Potsdam University, Building 14, Karl-Liebknecht-Straße 24-25, 14476 Potsdam, Germany; 3 ETH Zurich, Stampfenbachstrasse 69, 8092 Zurich, Switzerland; 4 Flinders University Institute for Mental Health and Wellbeing, College of Education, Psychology and Social Work, Flinders University, Sturt Rd, Bedford Park, SA 5042 Australia; 5 University of Leeds, University Rd, Woodhouse, Leeds, LS2 9JT UK; 6 University of Hull, Hull, UK

**Keywords:** Eye contact, Communicative intent, Human–AI interaction, Artificial intelligence, Joint attention, Intentional stance, Psychology, Human behaviour

## Abstract

Communication with artificial agents, such as virtual characters and social robots, is becoming more prevalent, making it crucial to understand how their behaviours can best support social interaction. Eye gaze is a key communicative behaviour, as it signals attention and intentions. Prior research shows that perceiving an agent as sentient affects how its gaze is interpreted. This study examined how such beliefs affect the interpretation of gaze as a signal of communicative intent. In a semi-interactive online task, 160 participants viewed a virtual agent exhibiting dynamic gaze sequences. Each trial varied whether eye contact occurred and whether the agent looked at the same object twice. Participants judged whether the agent was requesting help or merely inspecting the object. Beliefs about the agent’s sentience (human- or AI-controlled) were also manipulated. Results showed that when gaze cues were ambiguous, participants were more likely to ascribe communicative intent if they believed the agent was human-controlled compared to when they believed the agent was AI-controlled. Subjective ratings also indicated a general preference for human-controlled agents. These findings underscore the influence of user expectations on interpreting gaze in artificial agents.

## Introduction

Virtual and artificial agents, including animated characters in virtual reality (VR) and social robots, are becoming increasingly prevalent across various sectors, such as healthcare^1^, education^2^, the service industry^3^, and marketing^4^. Specifically, artificial intelligence (AI) is transforming daily life, enhancing human health, safety, and productivity^5–8^. Interactions with these agents are increasingly resembling human–human interactions^9^, as many of these agents now display human-like features and multimodal behaviours such as social gaze displays, facial expressions, and gestures^10,11^. This perceived humanlikeness can lead the user to engage in ‘anthropomorphism’—the attribution of human-like traits and intentions^12–16^. However, people vary in their tendency to engage in anthropomorphism^17^, and this variation is further amplified by the differences in degree of humanlikeness exhibited by virtual and artificial agents^18^. Human-like agent features (e.g., appearance, behaviours) and user predispositions (e.g., anthropomorphism tendencies, beliefs about agents) can influence human–machine interactions in a bottom-up (agent-driven) and top-down (user-driven) manner respectively^10,19–24^. This influence can be positive or negative, depending on the context. For instance, while human-like appearance can foster trust and acceptance of *social* robots^20,21,25–27^, it may negatively impact the perceived reliability and attention allocation for *industrial* robots^28^. Understanding how the influence of an agent’s appearance and behaviour interacts with or is overwritten by our beliefs about agents (e.g., that they are artificially controlled), is critical to informing how we design and position agents in a way that promotes effective communication with, and acceptance by, humans.

A key behavioural feature of human-like artificial agents is social gaze, which also plays a crucial role in *human–human* interactions. Eye gaze in human–human interactions is a particularly rich source of social information^29^, providing constant and rapid information about others’ visual perspectives and intentions^29–31^. As such, eye gaze information is often used to signal and identify opportunities for ‘joint attention’—the coordination of attention and actions with others^32^. Joint attention plays a crucial role in the development of language and social cognition in children, particularly, in understanding another person’s unobservable mental states, such as intentions, beliefs, desires^33,34^, and remains essential for collaborative interactions throughout life^35^. Successfully achieving joint attention activates regions in the social brain network associated with attributing mental states to another person (‘mentalising’)^36^.

In *human–machine* interactions, eye gaze also plays a crucial role^37–40^. Recent studies by Caruana and colleagues have explored how gaze behaviour of artificial agents, including the frequency, contextual sequence, and timing of eye contact within dynamic eye movements, influences both the likelihood and certainty (i.e., speed) with which people interpret an agent as signalling communicative intent^41^. In a paradigm by Hechler and colleagues^42^, participants engaged in a semi-interactive task with an on-screen agent that shifted its gaze three times in each trial, inspecting three different objects (see their OSF page for details on the design, data, and analysis: https://osf.io/6uyhr/). The task was to decide whether the agent’s gaze behaviour signalled a request for one of the objects (signalling communicative intent) or whether the agent was privately inspecting the objects (without communicative intent). The study manipulated across trials whether the agent displayed eye contact and made repeated averted gaze shifts at the same object. Results showed that eye contact and repeated averted gaze shifts to the same object independently and additively increased ascribed communicative intent (henceforth ‘**Eye Contact**’ and ‘**Repeated Gaze**’ effects). Eye contact was the more potent signal of the two, but the strongest effect occurred when both features were present together. While these effects generalised across human- and robot-looking agents, the study did not control for whether participants *believed* that the agent’s behaviour was human- or machine-generated.

As users interact with increasingly human-like agents, their belief about an agent’s humanness—in particular, the assumption that they have human-like mental states and capabilities (i.e., *intentional stance belief*)^24^—becomes an important factor in shaping the human–machine interaction^43^. Adopting an intentional stance, that is, assuming an entity possesses intentional qualities and agency^44–46^, may be an effective way for non-expert users to interpret and predict the behaviour of artificial agents, given their complexity^47^. For instance, users may perceive a virtual assistant as ‘intending’ to optimise their schedule instead of trying to understand the underlying algorithms and code. This focus on the assistant’s ‘intentions’ rather than its technical complexity may simplify the interaction. When people adopt an intentional stance, they may perceive artificial agents as more trustworthy^48^ or engaging^49^ and benefit from increased communication quality and collaboration^24,50^. Specifically, the adoption of an intentional stance may modulate how people respond to gaze cues as they tend to attribute greater relevance to gaze cues when they believe them to be indicative of the intentions of a sentient being^19,40,51–54^. Gaze-cueing studies could show how believing screen-based virtual agents or physical robots to be human- instead of computer-controlled may modify behavioural strategies for social coordination, including increased degrees to which gaze is followed^52,53,55^, more positive subjective experiences^55^, and modulated neural processing of gaze shifts that signal the achievement or avoidance of joint attention^53,56,57^. For instance, Abubshait and Wykowska^51^ showed that attention orienting to robot gaze-cues was positively related to how much intention was attributed to the robot. Thus, the perceived physical humanness of an agent and the belief that changes in gaze direction are intentional rather than unintentional may impact the social relevance of gaze and, thus, increase or decrease the likelihood of following it^58,59^. While previous research has primarily compared the discrete categories human- and computer-controlled agents, it remains unclear whether these effects persist when comparing human- and *AI*-controlled agents. In recent years, significant advancements in AI may have elevated public expectations regarding the intelligence and capabilities of AI-controlled agents. Consequently, the perceived difference between humans and artificial agents in basic social interactions may be narrowing, with the expectation that these computer systems can now adapt to different contexts and, consequently, increase attributions of intelligence, sentience and consciousness^60^. Research has shown that the tendency to attribute mental states decreases from humans to robots and then to computers^61^. Given that AI is narrowing the gap between human and artificial agents, it is important to understand where AI-controlled agents fit within this hierarchy.

The current study builds on the experimental design of Caruana and colleagues^41,42^ to investigate how agent gaze behaviour and user beliefs about the agent’s agency shape the interpretation of eye gaze as communicative. Understanding the role of the intentional stance in ascribing communicative intent is essential for defining the conditions under which people are likely to be most receptive to the social behaviours of artificial agents. In our task, the agent performed short gaze sequences toward three objects. We manipulated in each trial whether the agent displayed eye contact and whether or not the agent repeatedly looked at the same object in the gaze sequence. Participants used their keyboard to decide on each trial whether to ‘give’ one of the objects to the agent. We anticipated replicating previous findings on the effects of gaze dynamics on ascriptions of communicative intent. Specifically, based on Hechler and colleagues^42^, we expected participants to most likely ascribe communicative intent when the agent made eye contact and repeated averted gaze displays (e.g., object 1, eye contact, object 1), followed by eye contact without gaze repetition (e.g., object 1, eye contact, object 2), followed by gaze repetition without eye contact (e.g., object 1, object 2, object 1), and, finally, no eye contact or gaze repetition (e.g., object 1, object 3, object 2). These predictions are consistent with previous findings showing that communicative intent is most strongly inferred when eye contact precedes an object-directed gaze shift, whereas private intentions are inferred primarily from gaze toward objects alone^62^. These findings highlight eye contact as a particularly powerful ostensive cue for communication and suggest that combining eye contact with additional gaze signals enhances ascriptions of communicativeness. Consistent with this, Senju and colleagues^63^ showed that six-month-old infants follow gaze only when preceded by ostensive cues such as direct gaze or infant-directed speech, underscoring the critical role of ostension in triggering communicative interpretations. From a theoretical perspective, these observations are in line with the proposal that human communication is supported by a dedicated system of *natural pedagogy*^64,65^. According to this framework, ostensive signals such as direct gaze serve to indicate that communication is intended, induce referential expectations, and bias observers to interpret subsequent signals as conveying generalisable information. Thus, ostensive gaze cues can be understood not merely as attention-grabbing, but as part of an evolved communicative system that scaffolds the attribution of communicative intent.

Importantly, evidence from infant gaze-following studies also suggests that communicative attributions can arise from attention-grabbing actions that are not conventionally ostensive. Six-month-old infants have been demonstrated to follow gaze not only after direct eye contact but also after non-ostensive yet salient cues such as shivering or nodding^66,67^. These findings challenge the assumption that ostensive signals are uniquely required for gaze following and instead support the view that social attention mechanisms broadly facilitate communicative interpretations. In addition, Senju and colleagues^68^ provided converging evidence that nine-month-old infants are sensitive to the referential relation between gaze direction and object location, but only when the gaze shift is preceded by direct eye contact. Their findings underscore that eye contact acts as a communicative signal enabling infants to treat subsequent gaze as referential, and that repeated gaze shifts further strengthen such encoding. Together, these studies align with our prediction that repeated gaze shifts, even in the absence of eye contact, would still elicit intermediate levels of ascribed communicative intent, whereas eye contact combined with gaze shifts would maximise attributions of communicativeness.

We further expected faster reaction times for ‘give’ than ‘no-give’ responses in the more communicative conditions, and the inverse effect in the less communicative conditions. This pattern would indicate greater certainty of communication when eye contact and gaze repetition cues were displayed and greater certainty of no communication when both cues were absent. Previous work by Jording and colleagues showed that participants base their interpretation of gaze as communicative or private on the occurrence of eye contact and the location of the subsequent gaze shift^62^ and are slower and less coherent in inferring interactivity from gaze patterns when these are more ambiguous^69^.

Critically, for the current study, we anticipated that the bottom-up effects of gaze dynamics on evaluations of communicative intent would be modulated by top-down beliefs about human agency. To test this hypothesis, we manipulated within-subjects whether participants believed that the eye movements of the artificial agent were modelled on human- or AI-generated data. We expected that perceptual signals of communicative intent (e.g., eye contact) would have greater impact when perceived as reflecting the intentional behaviours of a human agent rather than an AI system.

## Results

In this semi-interactive task, participants observed a virtual human-like agent performing short gaze sequences toward one of three objects, embedded in a collaborative block-construction scenario. Participants judged whether the agent’s gaze behaviour indicated a communicative request (‘give’) or a private visual search (‘no-give’). Reaction times were recorded to index response certainty. On each trial, two gaze parameters were manipulated *within-subjects*: (1) **Eye Contact**: whether the agent made direct gaze toward the participant during the middle gaze shift and (2) **Repeated Gaze**: whether the agent looked at the same object before and after the middle gaze shift. This 2x2 manipulation produced four distinct gaze conditions: (1) **No Eye Contact + No Repeated Gaze**; (2) **No Eye Contact + Repeated Gaze**; (3) **Eye Contact + No Repeated Gaze**; and (4) **Eye Contact + Repeated Gaze**. We also implemented a belief manipulation that varied participants’ assumptions about the agent’s control source (**Belief**): in one block, they were told the gaze behaviour was based on human eye-movement data (**Human-Belief**); in the other, it was said to be AI-generated (**AI-Belief**). Block order was counterbalanced across participants. This design allowed us to assess main and interactive effects of *bottom-up* gaze cues (**Eye Contact**, **Repeated Gaze**) and *top-down* beliefs (**Belief**) on the ascription of communicative intent.

### Behavioural measures

#### ‘Give’ frequencies

We examined whether participants’ **Belief** influenced the tendency to ‘give’ (i.e., ascribe communicative intent) across the gaze conditions (**Eye Contact** and **Repeated Gaze**). The final model after model selection accounted for within-subject and between-trial variability with random intercepts for subject, subject:**Eye Contact**, subject:**Repeated Gaze**, subject:**Belief**, subject:**Eye Contact**:**Repeated Gaze**, subject:**Eye Contact**:**Belief**, subject:**Repeated Gaze**:**Belief**, subject:**Eye Contact**:**Repeated Gaze**:**Belief**, and trial. Descriptive statistics for both behavioural measures are summarised by condition in Table 1. The random effect components of the model explained a significant amount of variance, as indicated by a marginal $R^2$ of .31 (which reflects the variance explained by the fixed effects alone) and a conditional $R^2$ of .83 (which includes the variance explained by both the fixed and random effects).

**Table 1:** Descriptive statistics by Gaze Condition, Belief, and Response.

Gaze condition	Belief	Give frequency %	Give RT	No-Give RT
Eye Contact, No Repeated Gaze	AI	76.6 (31.6)	0.83 (0.51)	0.95 (0.54)
	Human	77.31 (31.99)	0.85 (0.51)	0.88 (0.55)
Eye Contact, Repeated Gaze	AI	98.28 (3.9)	0.63 (0.35)	0.96 (0.58)
	Human	97.25 (6.62)	0.64 (0.35)	1.12 (0.64)
No Eye Contact, No Repeated Gaze	AI	41.18 (38.04)	0.84 (0.5)	0.75 (0.42)
	Human	39.52 (37.67)	0.87 (0.5)	0.73 (0.42)
No Eye Contact, Repeated Gaze	AI	59.92 (37.95)	0.81 (0.46)	0.74 (0.46)
	Human	61.44 (37.33)	0.83 (0.5)	0.73 (0.44)

’Give’ frequencies are summarised as the percentage of trials that participants responded by giving a block to the agent. Reaction times are summarised by response. Means and Standard deviations are reported in the format ‘M (SD)’

**Table 2:** Exposure groups.

Gender	Number of participants		Age		CATI total score		ATTARI total score	
	n	%	Mean	SD	Mean	SD	Mean	SD
Female	64	51.2	41	13	106.34	28.97	166.36	39.56
Male	61	48.8	38	12	102.58	29.36	171.91	42.08
Total	125	100.0	40	1	104.46	0.28	169.14	1.78

**Fig. 1:**
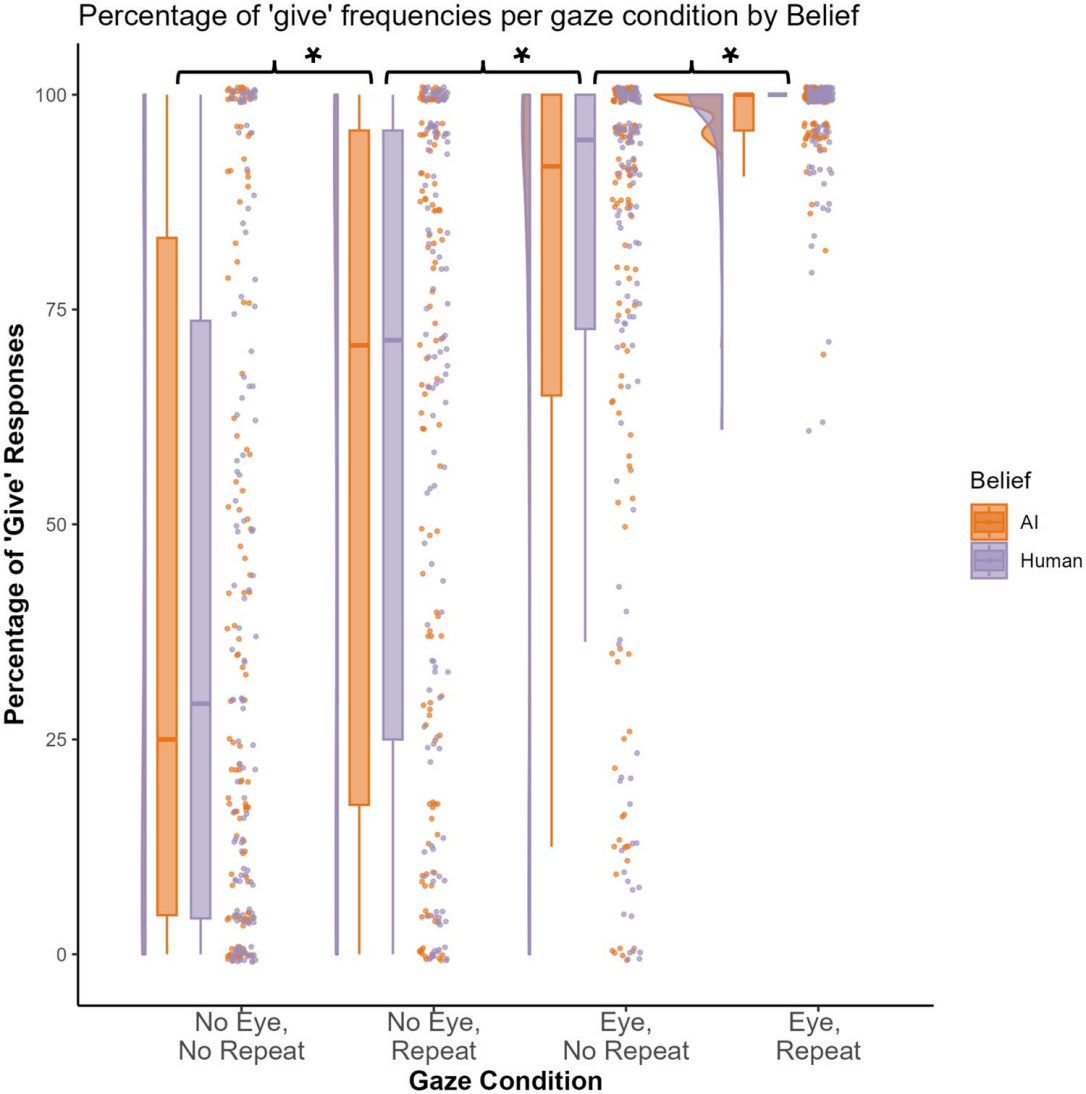
‘Give’ frequencies per gaze condition. AI Belief data is displayed in orange and Human Belief data in violet. The condition labels are abbreviated as follows: ‘No Eye Contact, No Repeated Gaze’ = ‘No Eye, No Repeat’, ‘No Eye Contact, Repeated Gaze’ = ‘No Eye, Repeat’, ‘Eye Contact, No Repeated Gaze’ = ‘Eye, No Repeat’, ‘Eye Contact, Repeated Gaze’ = ‘Eye, Repeat’. Significant differences from pairwise comparisons between each Eye Contact+Repeated Gaze, averaged across Belief groups condition are indicated with an asterisk.

We replicated, as expected, the significant main effects of **Eye Contact** ($\beta$ = −1.99, *SE* = 0.17, *p* < .001) and **Repeated Gaze** ($\beta$ = −1.31, *SE* = 0.14, *p* < .001) as well the significant interaction between **Eye Contact** and **Repeated Gaze** ($\beta$ = 0.45, *SE* = 0.11, *p* < .001). Participants were more likely to ‘give’ when the agent displayed eye contact (*M* = 87.36, *SD* = 25.01) than when it displayed no eye contact (*M* = 50.51, *SD* = 39); and when it repeated averted gaze displays (*M* = 79.22, *SD* = 32.62) than when it displayed gaze shifts to unique locations (*M* = 58.65, *SD* = 39.38). Critically, the likelihood to ‘give’ was higher when the agent displayed eye contact together with a repeated averted gaze display. Pairwise comparisons revealed significant differences in ‘give’ frequencies between all four gaze conditions. People were significantly more likely to ‘give’ when the agent displayed eye contact and repeated averted gaze displays (**Eye Contact+Repeated Gaze**: *M* = 97.77, *SD* = 5.45) than when averted gaze displays were unique (**Eye Contact+No Repeated Gaze**: *M* = 76.95, *SD* = 31.73; Estimate = −3.52, *SE* = 0.39, 95% CI [−4.46, −2.58], z = −8.93, *p* < .001); when the agent displayed eye contact with unique averted gaze displays than no eye contact and repeated gaze (**No Eye Contact+Repeated Gaze**: *M* = 60.68, *SD* = 37.58; Estimate = 1.36, *SE* = 0.4, 95% CI [0.29, 2.43], z = −8.93, *p* < .001); and when the agent displayed no eye contact and repeated gaze than no eye contact with unique gaze displays (**No Eye Contact+No Repeated Gaze**: *M* = 40.35, *SD* = 37.79; Estimate = −1.7, *SE* = 0.32, 95% CI [−2.53, −0.88], z = −5.32, *p* < .001). All other comparisons between conditions were also significant (all *p*s < .001; see supplementary material on the OSF project page for a detailed report: https://osf.io/wcm75). The pattern of give frequencies across the four gaze conditions and between belief conditions are visualised in Fig. 1 (see the 03_Plots.Rmd output for a visualisation of the two blocks separately).

The primary aim of this study was to investigate whether participants’ **Belief** about the intentionality of the agent influenced their tendency to ‘give’. While we found no evidence for a significant main effect of **Belief** (*p* = .300), there was evidence for a three-way interaction between **Belief**, **Eye Contact**, and **Repeated Gaze** ($\beta$ = 0.12, *SE* = 0.04, *p* = .003), indicating higher ‘give’ frequencies in the ambiguous conditions when participants believed to observe human- (**No Eye Contact+Repeated Gaze**: *M* = 61.44, *SD* = 37.33; **Eye Contact+No Repeated Gaze**: *M* = 77.31, *SD* = 31.99) rather than AI-modelled behaviour (**No Eye Contact+Repeated Gaze**: *M* = 59.92, *SD* = 37.95; **Eye Contact+No Repeated Gaze**: *M* = 76.6, *SD* = 31.6). However, follow-up pairwise comparisons between **Belief** conditions for each individual gaze condition showed no significant differences in ‘give’ frequencies (all *p*s >.37). To examine the **Belief x Eye Contact x Repeated Gaze** interaction further, we explored whether the absence of significant pairwise comparisons between **Belief** conditions was due to the averaging of response frequencies across block 1 and block 2 of the experiment; across which the belief order was counterbalanced. To this end, we examined whether there was any evidence for a different pattern of results when examining the block 1 and 2 datasets separately; essentially treating the **Belief** factor as a between-subjects factor, and removing the potential influence of order effects on the **Belief** manipulation. We found that the **Belief x Eye Contact x Repeated Gaze** interaction was neither significant for block 1 (*p* = .123) nor block 2 (*p* = .243).

#### Reaction times

**Fig. 2:**
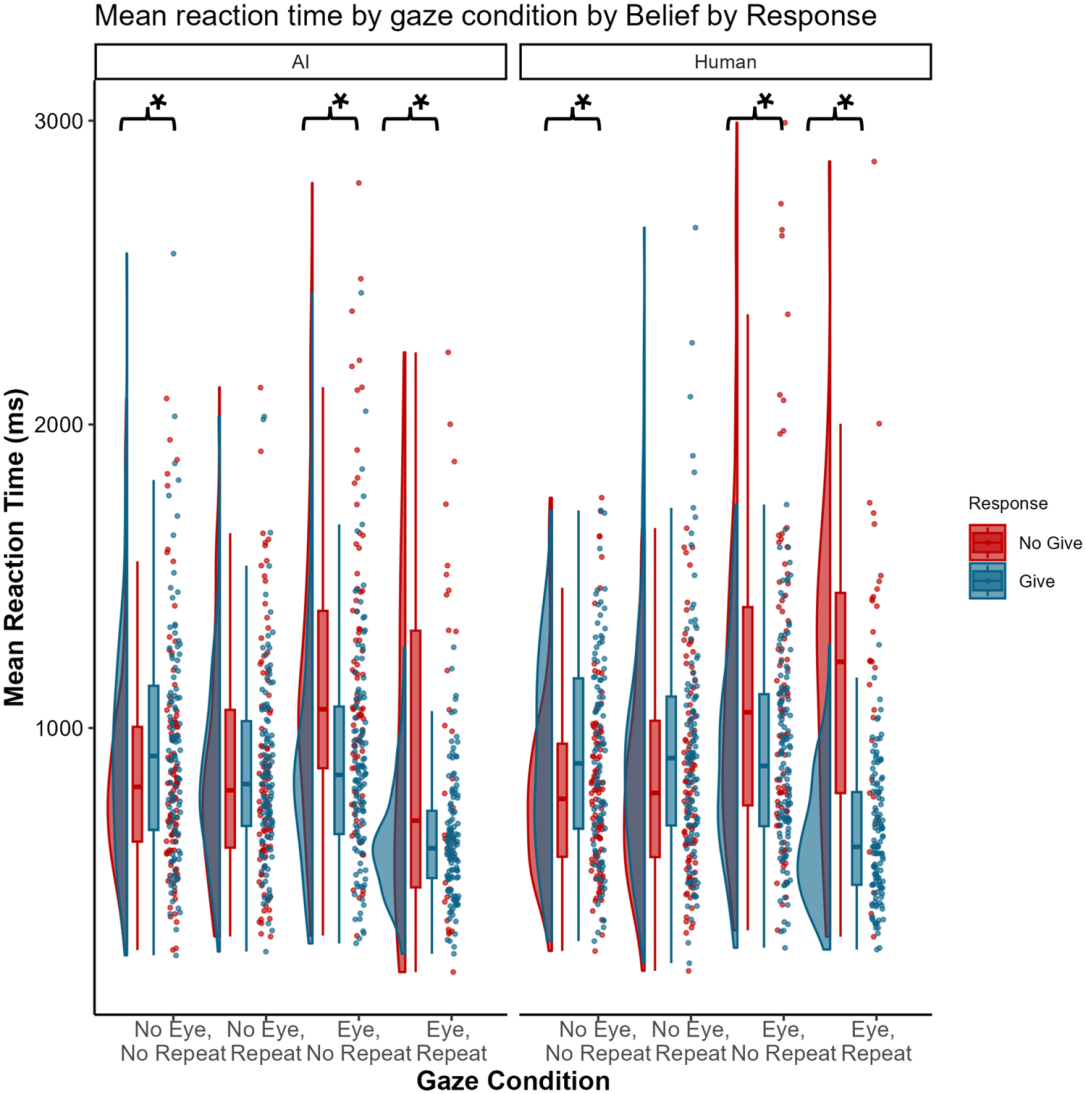
Reaction times per gaze condition. AI Belief data is displayed on the left and Human Belief data on the right. ‘No-give’ response data is displayed in red and ‘give’ response data in blue. The condition labels are abbreviated as follows: ‘No Eye Contact, No Repeated Gaze’ = ‘No Eye, No Repeat’, ‘No Eye Contact, Repeated Gaze’ = ‘No Eye, Repeat’, ‘Eye Contact, No Repeated Gaze’ = ‘Eye, No Repeat’, ‘Eye Contact, Repeated Gaze’ = ‘Eye, Repeat’. Significant differences from pairwise comparisons between Response conditions for each Eye Contact+Repeated Gaze condition, separately for each Group are indicated with an asterisk.

We also investigated the influence of the above factors on RTs for a **Give** or **No-Give Response**. The frequency analysis reported above evaluated the influence on the *likelihood* of ascribing communicative intent, whereas the RT analysis evaluates *certainty/ambiguity* in the ascription of communicative intent. The final model after model selection included random intercepts for subject, subject:**Give Response**, subject:**Belief**, subject:**Eye Contact**:**Repeated Gaze**, subject:**Give Response**:**Eye Contact**, subject:**Give Response**:**Repeated Gaze**, subject:**Give Response**:**Belief**, subject:**Give Response**:**Eye Contact**:**Repeated Gaze**:**Belief**, and trial. Descriptive statistics for RTs are summarised by condition in Table 1 and visualised in Fig. 2. The random effect parameters in the model explained a significant amount of variance, as reflected in both the marginal $R^2$ value (.06) and conditional $R^2$ value (.45).

Once more, we replicated the findings reported by Hechler and colleagues^42^. Our analysis showed evidence for a **Response x Eye Contact** interaction ($\beta$ = −0.106, *SE* = 0.01, *p* < .001; $\eta _p$ = 0.260, 95% CI [0.200, 1.000]); and a **Response x Repeated Gaze** interaction ($\beta$ = −0.05, *SE* = 0.01, *p* < .001; $\eta _p$ = 0.076, 95% CI [0.041, 1.000]). We also found evidence for a **Response x Eye Contact x Repeated Gaze** interaction ($\beta$ = 0.026, *SE* = 0.01, *p* < .001; $\eta _p$ = 0.030, 95% CI [0.007, 1.000]). This three-way interaction suggested that, in the most communicative condition, (i.e., **Eye Contact+Repeated Gaze**) participants were significantly faster to ‘give’ (*M* = 633.33, *SD* = 199.36) than ‘not give’ (*M* = 1045.06, *SD* = 550.72; Estimate = 0.48, *SE* = 0.06, 95% CI [0.36, 0.57], t = 8.32, *p* < .001); and, in the least communicative condition (i.e., **No Eye Contact+No Repeated Gaze**), participants were significantly faster to ‘not give’ (*M* = 823.51, *SD* = 329.64) than ‘give’ (*M* = 928.49, *SD* = 364.28; Estimate = −0.15, *SE* = 0.03, 95% CI [−0.23, −0.05], t = −4.3, *p* < .001). For the more ambiguous conditions, differences in RTs between ‘give’ and ‘no-give’ responses were smaller. When the agent made no eye contact but repeated the averted gaze display, RTs were not found to differ (**No Eye Contact+Repeated Gaze**: *p* = .675). However, when the agent displayed eye contact but did not repeat the gaze, pairwise comparisons between ‘give’ and ‘no-give’ responses revealed significantly shorter ‘give’ (**Give Response+Eye Contact+No Repeated Gaze**: *M* = 897.86, *SD* = 345.09) than ‘not give’ response RTs (**No Give Response+Eye Contact+No Repeated Gaze**: *M* = 1153.16, *SD* = 523.73; Estimate = 0.17, *SE* = 0.04, 95% CI [0.07, 0.25], t = 4.82, *p* < .001).

Finally, we found evidence for a **Response x Belief x Eye Contact x Repeated Gaze** interaction ($\beta$ = −0.015, *SE* = 0.01, *p* = .026; $\eta _p$ = 0.002, 95% CI [0.000, 1.000]). Pairwise comparisons of this four-way interaction only confirmed, for both groups, the same **Response x Eye Contact x Repeated Gaze** interaction reported above, since comparisons between **Belief** conditions within each of the gaze conditions were not significant (all *p*s >.70).

### Agent perception

We explored whether people formed different expectations and perceptions of the agent stimulus depending on their intentional stance. Participants rated their expectations regarding the agent and the upcoming interaction before each **Belief** block and made similar retrospective ratings on the agent and the interaction after each block. The results of a cumulative link model analysis revealed that, before each **Belief** block, participants had significantly lower ratings of the AI- than the human-modelled agent with respect to their expectation that the agent would be (1) a good communicator ($\beta$ = 0.96, *SE* = 0.24, *z* = 3.99, *p* < .001); enjoyable to interact with ($\beta$ = 0.5, *SE* = 0.23, *z* = 2.14, *p* = .033); and (3) easy to interact with ($\beta$ = 0.79, *SE* = 0.24, *z* = 3.37, *p* < .001). After each block, participants provided the same ratings about the agent under each **Belief** condition. Retrospective judgements did not significantly differ between **Belief** conditions (all *p*s >.607).

However, when participants compared the two agents on a single scale after both blocks, they rated the human as more preferable than the AI agent across several dimensions. Specifically, we conducted Wilcoxon Rank-Sum tests to determine whether participants’ preferences for the human agent were significantly greater than the neutral midpoint of the scale. This indicated that participants significantly preferred the human over the AI with respect to its communication (*M* = 3.18, *SD* = 1.03), V = 6928, *p* = .002), pleasantness (*M* = 3.16, *SD* = 0.78), V = 1548, *p* = .002), and naturalness (*M* = 3.42, *SD* = 0.88), V = 5629, *p* < .001).

**Fig. 3:**
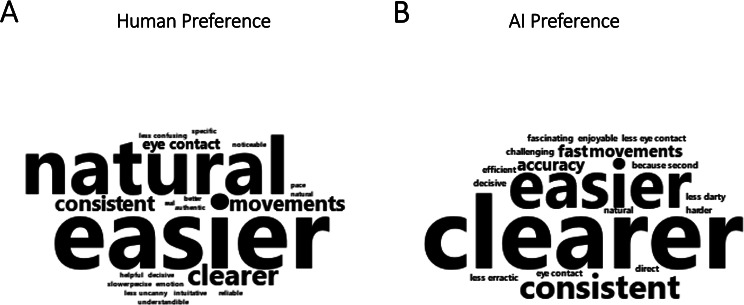
Free-text responses to the question ‘Which partner did you prefer? Why?’. Responses of participants who preferred the human are shown in (**A**) and responses of participants who preferred the AI in (**B**). Phrases that appeared more frequently are displayed larger.

58 participants indicated, with an overall preference rating above 3, a preference for the agent in the **Human-Belief** condition, 38 participants indicated, with a rating below 3, a preference for the AI agent, and 38 participants indicated, with a rating of 3, no preference. While the variation in these preferences for either agent was not significant (*M* = 3.14, *SD* = 1.25), V = 9378, *p* = .063), free-text responses indicated that participants who preferred the human mostly did so because they perceived this agent as more “natural” (*n* = 11), whereas participants who preferred the AI mostly did so because they perceived this agent as a clearer communicator (*n* = 12), as can be seen in the word clouds in Fig. 3. A shared prominent reason in both groups was that the respective agent was “easier” (Human: (*n* = 14; AI: *n* = 8).

## Discussion

The current study examined how beliefs about an artificial agent’s intentionality shape the evaluation of gaze behaviour as signals of communicative intent—an essential process for recognising and responding to communication opportunities (e.g., joint attention). As expected, we replicated the pattern observed by Hechler and colleagues^42^, with individual and cumulative effects of **Eye Contact** and **Repeated Gaze** on the ascription of communicative intent. Such gaze dynamics need not uniquely signal communicativeness and could also reflect personal interest or uncertainty; however, in our task they biased participants toward ascribing communicative intent. Importantly, consistent with our previous work, we do not argue that repeated gaze alone is communicative. Rather, we suggest that it likely functions as a cue to relevance, which, when combined with the communicative potential of eye contact, promotes the impression of an intentional and relevant communicative gaze behaviour. Participants were more likely to ‘give’ when the agent displayed eye contact compared to when it did not, and when it displayed repeated averted gaze shifts to the same location than to unique locations. Critically, ascriptions of communicative intent were strongest—in terms of response frequency and speed—when eye contact and gaze repetition cues were presented together. This replication aligns with evidence from developmental psychology research showing that eye contact is a critical ostensive signal for eliciting gaze following and communicative interpretations in infancy^63,68^, and with studies demonstrating that non-ostensive but attention-grabbing cues (e.g., shivering, nodding) can also trigger gaze following^66,67^. Our RT data further supports this conclusion. We found that the absence of eye contact or repeated averted gaze displays independently increased the certainty to ‘not give’ and their presence increased the certainty to ‘give’. At the same time, conditions that involved partial signals of communicative intent (e.g., repeated averted gaze displays without eye contact) led to greater variability in ‘give’ responses and less differentiation in RTs to ‘give’ or ‘not give’, suggesting greater ambiguity in the evaluation of communicative intent. This interpretation also resonates with findings by Jording and colleagues^69^, where judgements of interactivity and corresponding reaction times varied systematically with the ambiguity of a virtual agent’s gaze signals. More ambiguous states (e.g., introspective gaze without any obvious attentional focus in the environment) elicited longer reaction times and less consistent judgements of interactivity, whereas clearer communicative signals (e.g., initiating joint attention) led to shorter reaction times and higher interactivity ratings. However, like Hechler and colleagues^42^, we also found faster ‘give’ than ‘no-give’ responses in the **Eye Contact + No Repeated Gaze** condition. In the original study, this unexpected pattern was mainly driven by heterogeneity induced by autistic participants, who constituted half of the sample^42^. However, given that the pattern was replicated in the current study, it may also reflect how eye contact and repeated averted gaze signal joint attention opportunities in different ways.

One possible explanation for different information provided by eye contact and gaze repetition, is that eye contact may directly signal communication *readiness* or *intention*, while repeated gaze may signal the *relevance* of one’s focus of attention and provide context to the communication content where joint attention is being signalled. Such an interpretation is in line with the ‘Relevance Theory’ proposed by Sperber and Wilson^70^, which posits that communication relies on the principle of relevance, where the signaller aims to be as relevant as possible, and the receiver seeks the most relevant meaning in order to minimise the cognitive costs of processing communicative signals. Within this framework, the concept of ostensive-inferential communication proposes that the signaller provides an ostensive gesture (e.g., eye gaze) that indicates an intention to convey a message (ostension). The receiver must then interpret this stimulus by inferring the communicator’s intention based on the context and/or their own knowledge. In the current study, eye contact serves as a clear ostensive signal, indicating the communicator’s intention to communicate, leading to increased certainty in the decision to ‘give’. Repeated averted gaze shifts, on the other hand, provide contextual information that influences the interpretation of the communicator’s intent. When these repeated gaze shifts occur without eye contact, this creates an ambiguous context that demands additional cognitive processing to infer the intention behind the signal. This would explain why we see more variability in RTs when deciding whether to ‘give’ when repeated gaze is present but eye contact is not. In summary, eye contact acts as a strong ostensive signal, signalling *communicativeness*, while repeated averted gaze shifts may provide contextual cues that can either clarify or obscure the intended message by signalling *relevance*. This distinction is important, as people often follow gaze cues even when the gazer is not intentionally guiding them^71^. Given that joint attention is sometimes achieved when the initiator does not intend to communicate their focus of attention (e.g., looking at the target in a game of ‘eye spy’ or looking at the clock when bored in conversation), it would also be valuable to examine evaluations of gaze as signalling intentional and unintentional communication and joint attention opportunities. This would require a modified task that does not explicitly demand evaluations of whether an agent is intending to communicate, but rather whether the agent is attending to something of interest or relevance. Interactive eye tracking studies of joint attention, similar to those conducted by Caruana and colleagues^38,72^ may also be adapted to examine how eye contact and gaze repetition influence spontaneous joint attention in unconstrained interactions, thus removing the need for explicit judgements altogether. This line of research would shed light on how artificial agents can strategically use gaze behaviour to facilitate intuitive social coordination with humans.

The primary focus of the current study was to determine how beliefs about the intentionality of the agent (AI vs. human) influenced the evaluation of its gaze behaviour. We found evidence for a significant **Belief x Eye Contact x Repeated Gaze** interaction, with higher ‘give’ frequencies in the ambiguous conditions when participants believed to observe human- rather than AI-modelled behaviour. However, we found no evidence for specific **Belief** effects within each gaze condition when examined using follow-up pairwise comparisons. This may be explained by similar *implicit* beliefs about the agent’s intentionality being held across the two **Belief** blocks. While participants stated after the experiment to have explicitly believed that the behaviour they observed was human- or AI-modelled respectively, they might have implicitly adopted an intentional stance towards both the human and the AI agent. This aligns with the notion that the intentional stance is the default for understanding artificial agents^50^ and it might reflect how the gap between perceptions of human and AI agents is decreasing as (1) AI technologies become more advanced, and (2) people become more exposed to these AI technologies. For instance, a longitudinal study on public opinion and human–AI interaction conducted in the US reported that, while only a small minority of participants perceived current AIs as sentient, a significant majority believed AI could achieve sentience in the future or were uncertain about its potential^73^. The authors concluded that perceptions of mind can enhance, diminish, or add complexity to well-established human-computer interaction dynamics. Together with the current findings, this suggests that, despite explicit beliefs about the nature of the agent, implicit assumptions about intentionality may be influencing participants’ responses, highlighting the need for further research into the cognitive processes underlying human–AI interactions.

It is also possible that the observation of gaze dynamics—and in particular the establishment of eye contact—may have contributed to the implicit adoption of an intentional stance across both **Belief** blocks, even in the condition where people explicitly believed the agent was AI-modelled. Low-level perceptual gaze cues may have automatically engaged mentalising mechanisms, leading participants to infer intentions. Such an interpretation is consistent with theories and associated evidence that suggest that observing or experiencing eye contact automatically activates mentalising mechanisms in the brain, such as the Communicative Intention Detector^74^, Fast-Track Modulator^75^, and Watching Eyes account^76–78^. This interpretation is also in line with the Intentional Stance Model put forward by Wykowska and colleagues^53^, which proposes a bidirectional relationship between bottom-up sensory input and top-down cognitive processes. Gaze perception can recruit brain areas associated with social cognition (e.g., medial prefrontal cortex, temporoparietal junction), which can, in turn, influence the responsiveness of regions involved in gaze processing (e.g., superior temporal sulcus), and vice versa.

Finally, cognitive adjustments did not only influence the way participants interpreted gaze cues but also shaped subjective experiences of the interaction. Participants rated communication with the ‘human’ agent only *before* but not *after* the respective interaction as easier and more enjoyable than with the ‘AI’ agent. The lack of a difference in retrospective ratings may be due to the behaviour of the agent that was the same across blocks. Moreover, the **Belief** manipulation also influenced subjective experiences. Participants indicated that they significantly preferred the human partner with respect to their communication, pleasantness, and naturalness. This finding is in line with findings from Hechler and colleagues^79^. The authors found that believing an agent in VR-based social interactions to be human- instead of AI-controlled positively affected the subjective perception of both the agent and the interaction, even though this did not influence behavioural gaze use strategies. This is also consistent with earlier studies, which found human agent beliefs to result in more positive subjective experiences than artificial agent beliefs in studies that implemented more discreet belief manipulations (i.e., human- vs. computer-controlled)^55–57^. In the current study, there were not only **Belief** effects on subjective ratings but also differences in how participants explained their preferences for interacting with the “human-” and “AI- generated” agents. These differences, at least in communication-style preferences, may influence how **Belief** effects shape subjective experiences. Participants who, overall, preferred the human, explained that this was because the agent seemed more “natural”. Those participants who preferred the AI mostly explained that this was because the agent seemed to be a clearer communicator. Given that these perceptions could not be based on perceptual inputs—as the agent’s behaviour in both **Belief** conditions was the same—this could reflect the role that knowledge, expectations, and past experiences play on subjective experiences during human–AI interactions. Current AI systems vary widely in their capabilities, including computer vision, machine learning, and natural language processing^80^. Likewise, people vary in their experience with and knowledge about these systems^81^, which may impact how people approach artificial agents^82^. Future work should further evaluate how knowledge about, experience with, and attitudes towards AI shape the response to gaze cues in human–AI interactions.

### Limitations

The current study provides insights into how user beliefs about the intentionality of artificial agents shape the perception of gaze dynamics in *virtual* interactions. However, several limitations should be considered before generalising these findings to contexts involving physically embodied artificial agents (e.g., robots). First, while the virtual agent allowed for precise control over gaze cues and experimental conditions, it lacked multimodal cues that are typically present in real-world human–robot interactions, such as speech, facial expressions, and haptic feedback^83^. Thus, our scenario may not fully capture the complexities of more naturalistic interactions. For instance, it is possible that beliefs about human agency will have greater influence on other modalities of communication (e.g., facial expression interpretation, body posture or speech). Second, physical robots may elicit more positive social responses than virtual agents^48,84,85^. A review by Li and colleagues^86^ supports this pattern, although some findings suggest that social, rather than physical, presence is more critical for interaction^87^. As such, future work is needed to examine the extent to which the behavioural effects observed in the current study generalise to interactions with embodied artificial agents (i.e., physical robots).

In addition, we did not collect detailed demographic information such as participants’ educational background or cultural context, nor their broader experience with AI or robotics. These factors may shape how people approach artificial agents^81,81,88,89,89–92^ and thus limit the generalisability of our findings.

### Conclusion

Our study used a semi-interactive paradigm to investigate how beliefs about the human agency of an artificial agent impacts the evaluation of communicative intent from gaze behaviour. We directly tested whether the frequency of ‘give’ responses to gaze sequences was dependent on whether the agent’s behaviour was believed to be human- or AI-modelled. Our findings confirmed that eye contact plays a fundamental role in signalling communicative intent. Repeated gaze shifts also influenced evaluations of communicativeness. However, this may be a result of the contextual information repeated gaze cues provide regarding the relevance of gaze behaviour, rather than signalling communicative intent per se. The presence of both eye contact and repeated gaze maximised the likelihood of ascribing communicative intent. Importantly, we found numerical evidence that participants’ belief about the gaze behaviour being human- rather than AI-generated may increase the ascription of communicativeness when gaze cues were ambiguous—because they comprised mixed gaze signals (e.g., repeated gaze without eye contact)—suggesting that top-down agency beliefs inform evaluations in social conditions of uncertainty. Consistent with earlier findings, we also found beliefs to have robust influences on subjective social experiences, again highlighting how expectations of artificial agents are likely to shape interactions with artificial agents. Our findings have implications for the design of AI-driven communication systems, emphasising the need to consider both the behavioural cues of the agent and the evolving expectations that human users have about them.

## Methods

The current study builds upon a series of recent preregistered investigations that examined how the characteristics of perceived eye contact impact the interpretation of communicative intent. For a comprehensive theoretical rationale behind this research direction and an in-depth explanation of the original experimental design, please refer to the previous preregistration document available at: https://osf.io/w68ut/.

### Participants

We recruited 170 participants in total. However, following participant exclusion, based on our pre-registered protocol, we entered a data set comprising 125 participants into our final analyses ($M_{age}$ = 40; 61 identified as female; see Pre-Processing Data for a detailed summary of participant exclusion). The majority of people were born (31.62%) and resided in the United Kingdom at the time of completing the study (47.44%), with 43.59% identifying as white (see the supplementary material on the Open Science Framework project page (https://osf.io/wcm75 for detailed information on country of birth and residence, ethnicity, and student and employment status). Data was collected using the online data collection platform Prolific. Participants had to be at least 18 years old, be fluent in English, reside in Western English-speaking countries (United Kingdom, United States, Ireland, Australia, New Zealand, Canada), report no language-related disorders, and have a minimum Prolific approval rating of 95%. We compensated participants with £9.50 per hour for an approximate duration of 31 minutes. All participants provided written informed consent prior to completing the study, following the procedures approved by the Flinders University Human Research Ethics Committee (6804). All experiments were performed in accordance with relevant guidelines and regulations, in particular, with the Declaration of Helsinki (2013 revision).

### Experimental task and stimuli

We framed the task within a ‘collaborative’ context, instructing participants to ‘help’ an agent complete the construction of an off-screen block model. Participants were told that on each trial the agent was required to select a block to complete the model. In some trials, the block objects would be accessible to the agent, while in others the agent required the participant to ‘give’ them the required block. Participants observed the agent’s behaviour and decided whether to ‘give’ a block (i.e., because they interpreted the agent’s gaze behaviour as a communicative request) or not (i.e., because they interpreted the agent’s gaze behaviour as a private visual search) via a keyboard response. Full task instructions and experimental task code can be found on the corresponding OSF project page (https://osf.io/wcm75).

**Fig. 4:**
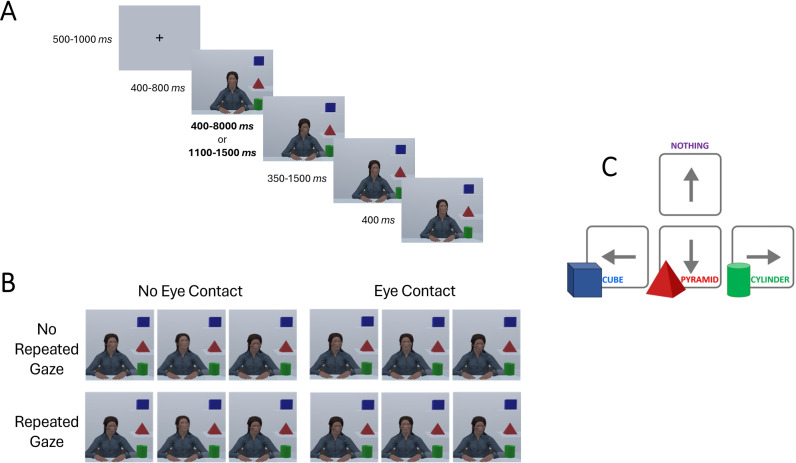
Trial sequence examples. In one block, participants completed the task assuming the data was modelled on human behaviour. In a second block, participants were led to believe they were seeing an agent reflecting AI-modelled behaviour. The blocks appeared in counterbalanced order across participants. Gaze duration of the second gaze shift, which displayed either eye contact or averted gaze depending on the gaze condition, summarised in (**B**), was counterbalanced as shown in (**A**). In each trial, participants decided whether to ‘give’ the agent one of the three blocks, or nothing at all, using the arrow keys on a standard keyboard (**C**).

Participants viewed a female agent directing the gaze towards one of three objects. We decided to use the same avatar throughout the study because previous work showed no evidence for avatar gender effects^79^ and we wanted to minimise adding additional factors in our within-subjects design. The avatar was created using Adobe Fuse CC (Beta Version 2014.3.14; San Jose, CA, USA) and was designed to be moderately realistic, with a slightly stylised appearance, and to appear ethnically ambiguous. This perception was validated in a separate sample of 47 participants ($M_{age}$ = 37.28, *SD* = 10.33; 31 identified as female)^39^. To simulate dynamic gaze behaviour, participants were presented with rapidly sequenced static images of the avatar, creating the impression of apparent motion and smooth gaze shifts. The second (middle) gaze shift was presented with two different counterbalanced durations, a short one ranging from 400–800 ms, and a long one ranging from 1100–1500 ms (see Fig. 4 A). These different durations were included, and controlled for, to increase the variability and ecological validity of gaze behaviour, and to enable future exploratory analyses on the potential role of gaze duration on ascriptions of communicative intent (not a focus of this current study). The avatar maintained a neutral facial expression to avoid introducing emotional cues or possible uncanny valley effects^93^.

In each trial, we manipulated within-subjects whether the avatar made eye contact with the participant or not in the second gaze shift (**Eye Contact**) and whether or not the avatar repeatedly looked at the same object before and after the second gaze shift (**Repeated Gaze**^41,42^ These two variables resulted in the following four different types of gaze stimuli (see also Fig. 4B):

1. **No Eye Contact+No Repeated Gaze** Three gaze shifts with no eye contact (i.e. the agent looks in different locations).
2. **No Eye Contact+Repeated Gaze** Averted gaze in between two other gaze shifts both directed at the same location (i.e. as condition 1 but the agent gazes at the same location in the first and third gaze shift).
3. **Eye Contact+No Repeated Gaze** Eye contact in between gaze shifts at different locations (i.e. as condition 1 but the agent makes eye contact in the second gaze shift).
4. **Eye Contact+Repeated Gaze** Eye contact, a gaze shift, and eye contact again (i.e. as condition 3 but the agent gazes at the same location in the first and third gaze shifts).

At the conclusion of each trial, participants decided whether to ‘give’ one of the objects via a keyboard response or to ‘not give’. At this stage, we presented an image of the response-key mapping as a visual reminder (see Fig. 4C).

### Procedure

The experiment was programmed in PsychoPy (v 2023.2.3) and deployed online via the Pavlovia platform. Participants accessed the task remotely on their own desktop or laptop computers. No specific restrictions or instructions were given regarding the testing environment (e.g., quiet surroundings). Prior to beginning the experimental task, participants were given the chance to practice the response-key mapping over the course of eight practice trials (two for each response key). During these practice trials, participants saw only the key-response mapping accompanied by a text prompt indicating the required response (e.g., “give nothing” or “give cylinder”). We provided feedback after each practice trial. The aim of this practice session was to minimise cognitive load during the task associated with response key mapping.

Participants completed the experimental task in two blocks. In one block, they were informed that the agent’s behaviour was modelled on real recordings of human eye movements. In the other block, participants were informed that the agent’s behaviour was generated by an AI system. Block order was counterbalanced across participants (**Human-Belief** vs. **AI-Belief**). Both of these blocks consisted of the same trial composition, with trials internally counterbalanced regarding the variation and sequence of gaze directions within each condition (24 trials for each experimental condition). Each block was divided into two sessions allowing participants to take two self-paced breaks. Participants completed 96 trials for each block (three sessions x 32 trials), and 192 trials in total (96 trials per block x two agents).

Before each of the two blocks, participants were informed whether their partner’s movements were “AI-modelled” or “human-modelled”. To check that participants attended to this belief manipulation, they were immediately promoted to indicate whether the agent in the upcoming block was human- or AI-modelled. Then, participants responded to questions about their expectations and saw another summary of the task instructions before the behavioural task began.

After completing a block, participants responded to questions on how they perceived their partner. The final block was followed by questions on task experience and partner perception. At the conclusion of the entire experimental task, participants completed a self-report measure of autistic traits^94^ and the Attitudes Towards Artificial Intelligence Scale (ATTARI-12)^95^. Finally, participants were debriefed and indicated whether they accepted the instruction that they completed the task with agents displaying human- and AI-modelled movements.

### Measures

**Behavioural measures** We collapsed responses across the four buttons into two **Response** categories (**Give**, **No-Give**). We calculated the proportion of trials in each condition with a **Give Response** to index the tendency of participants to interpret the agent’s behaviour as communicative (i.e., making a request). We also defined reaction times (RTs) as the time interval between the end of the trial and the participant’s button press. RTs were calculated for both **Give** and **No-Give Responses**, which we compared as an additional measure of response certainty/ambiguity within and across conditions. Specifically, shorter RTs indicated more certainty in the decision.

**Agent perception** We further explored whether top-down beliefs of the agent’s agency influenced subjective experiences during the task and evaluations of the agent. Previous work found these experiences to be influenced by a similar **Belief** manipulation^79^. To this end, participants completed throughout the experiment subjective rating and free-text impression questionnaires on partner perception and task experience created for this study (see supplementary material on the Open Science Framework project page (https://osf.io/wcm75). Specifically, before each **Belief** block, participants rated on 5-point Likert scale (“Not at all” - “Extremely”) their expectations regarding the agent’s communication clarity and enjoyment and ease of the interaction. After each **Belief** block, participants provided the same ratings retrospectively. Upon completing the entire experimental task, participants rated their preference for the AI or human on 5-point Likert scale (“AI was the best” - “Human was the best”) in terms of communication, pleasantness, and naturalness. Additionally, participants noted the most notable difference between the two agents in a free-text response and indicated their overall partner preference on a 5-point Likert scale, providing additional information in another free-text response. Finally, participants judged whether they accepted the instruction about the agents displaying human- and AI-modelled movements on a 10-point Likert scale and in a free-text response.

**Autistic traits** We also assessed participants’ autistic traits with the Comprehensive Autistic Traits Inventory (CATI)^94^ to characterise our sample. Autistic people often express difficulties perceiving or evaluating gaze information. While Hechler and colleagues found no evidence for impairments in autistic people in this task^42^, they reported, nevertheless, subtle differences in the evaluation of gaze information. The CATI is a reliable 42-item inventory that evaluates ‘sub-threshold’ autistic traits that may be present in individuals who do not meet the diagnostic criteria for autism. The questionnaire measures the trait dimensions *Social Interactions*, *Communication Difficulty*, *Social Camouflage*, *Repetitive Behaviours*, *Cognitive Rigidity*, and *Sensory Sensitivity*. Participants responded on a five-point Likert scale, with higher scores indicating a greater endorsement of autistic traits. Mean CATI scores are reported in Table 2.

**Attitudes towards AI** Finally, since the general set of attitudes towards artificial agents may influence the tendency to adopt an intentional stance, in that negative attitudes decrease the attribution of mental states^96^, we assessed attitudes towards AI with the *Attitudes Towards Artificial Intelligence Scale* (ATTARI-12).@stein_2024] The ATTARI is a recently developed measure designed to measure both positive and negative attitudes on a five-point Likert scale (1 = strongly disagree, 5 = strongly agree). The scale comprises general attitudes, considering cognitive, affective, and behavioural facets, with four items each, leading to twelve items in total. Mean ATTARI scores are reported in Table 2. The data was collected for characterising the sample and for future exploratory purposes. A Welch Two Sample *t*-test: was conducted to compare the total scores of participants who interacted first with the “AI-modelled” agent and participants who interacted first with the “human-modelled” data. The results indicated that participants who interacted first with the “AI-modelled” agent (*M* = 172.1, *SD* = 38.33) scored significantly higher than participants who interacted first with the “human-modelled” agent (*M* = 166.88, *SD* = 42.02), *t*(1040.26) = 2.12, *p* = .034. The data is available on the OSF framework https://osf.io/wcm75.

### Pre-processing data

We followed the pre-processing procedure specified in our preregistration (https://osf.io/wcm75). We initially collected, as pre-registered, 160 participants. At the end of the task, participants rated their acceptance of the instruction that they completed the task with agents displaying human- and AI-modelled movements on a scale of 1–10. We noticed that nine participants provided ratings less than ‘6’, indicating that they were sceptical of the manipulation. Hence, we recruited ten new participants (final sample of 170 participants) and we excluded those nine participants from all analyses. Next, we excluded nine participants from all analyses since they answered incorrectly to one of the two attention-check questions regarding whether the agent in the upcoming block was to be human- or AI-modelled.

While there were not objectively ‘correct’ or ‘incorrect’ responses in the experimental task, we assessed illogical responses that might indicate a random response style, including a ‘give’ response for an object that the agent never gazed towards. Note that this was not possible in the **No Eye Contact** condition in which the agent gazed at all three objects in these trials. We conducted analyses only on ‘logical’ responses. To begin, we removed 1493 trials (4.83%) with excessively short (i.e., < 150 ms) or long RTs (i.e., > 3000 ms) because these were likely pre-emptive or ‘guess’ responses. We then excluded seven participants whose rates of illogical answers exceeded 35%. Of the remaining participants, eight were excluded for having a rate of illogical answers that were more than 2.5 *SD* away from the sample mean. For the remaining participants, we checked for evidence of acquiescent response styles by examining the distribution of their ‘give’ responses across the three object options. Given the task’s counterbalancing of the agent’s gaze location, we expected ‘give’ responses to be equally distributed across the three ‘give’ response keys, with an *SD* of response frequency close to zero. We excluded five participants whose *SD*s were 2.5 *SD*s greater than the average *SD* observed across these three response options, as this suggested a marked preference to ‘give’ a particular object significantly more (or less) than the others. Following this, we removed all ‘illogical’ response trials from the remaining participants (2.38% of trials) and, then, four participants for whom we had to remove an excessive number of trials (number of retained trials < 2.5 *SD*s the sample mean). Lastly, three participants who were either too slow or too fast were removed (RTs < or > 2.5 *SD*s than the sample mean). The final dataset comprised 125 participants. The demographics of this final dataset can be found in Table 2.

### Analyses

All analyses followed our analysis plan pre-registered on the Open Science Framework (https://osf.io/wcm75) and were conducted using a custom R Markdown script with R Statistical Software (v4.5.0; R Core Team 2025), which can be found alongside all data on our OSF project page.

#### Behavioural measures

For the behavioural data (‘give’ frequencies and RTs), we used the same comprehensive data analysis approach as Caruana and colleagues^41^, to examine main effects and interactions related to **Eye Contact**, **Repeated Gaze**, **Belief**. For RT analyses, we also included the **Response** type (**Give**, **No-Give**) as an additional fixed effect, since the use of RT as an index of response certainty requires RTs to be examined as a function of the response provided. We transformed RTs using the box-cox *powerTransform* function from the car R package (v3.1.3)^97^ to address the often skewed RT distribution^98^, which may induce bias in the estimation of model parameters^99^.

Next, we applied linear mixed-effects (LME) and generalised linear mixed-effects (GLME) analyses on un-aggregated data, using the maximum likelihood estimation method within the lme4 R package (v1.1.37)^100^. This approach was chosen because we expected that some participants might not have consistently provided a ‘give’ or ‘no-give’ response within each condition, leading to bias when data is aggregated because aggregated RTs in one condition would be based on more observations than RTs in others. To ensure a maximal random factor structure, we followed the analysis pipeline recommended by Scandola and Tidoni^101^ incorporating model optimisation and implementation of complex random intercept (CRI) models. This pipeline entails the formulation of random effects structures, where intercepts are determined by several interacting factors, in cases where maximal models with random slopes for all effects cannot be reliably estimated using typical human datasets. Further details on the full and reduced models can be found in the supplementary material on the OSF project page (https://osf.io/wcm75). All *p*-values for the (G)LME models were estimated with the afex R package (v1.4.1)^102^, using a significance criterion of $\alpha$ < 0.05. We conducted post-hoc pairwise comparisons of individual conditions averaged across all other conditions using the emmeans R package (v1.11.0).@emmeans] For example, for the **Eye Contact x Repeated Gaze** interaction, we compared the **Eye Contact** and **No Eye Contact** conditions for both **No Repeated Gaze** and **Repeated Gaze**, averaging across all other conditions. This approach was applied to all significant interactions. Additionally, a Holm correction was used where appropriate to adjust contrasts for multiple comparisons.

#### Agent perception

To further investigate whether the perception of the agent differed between **Belief** conditions, we compared subjective ratings of the agent and the interaction made before and after each block between the **Belief** conditions using cumulative link models using the ordinal R package (v2023.12.4.1)^103^.

To test whether, after the interaction, participants were more likely to prefer the AI (i.e., preference scores closer to 1 on the scale) or human (i.e., a preference score closer to 5 on the scale) with respect to its communication, pleasantness, and naturalness, we performed separate Wilcoxon Rank-Sum tests, since Shapiro-Wilk tests for normality indicated that the data was non-normally distributed (all *p*s < .001).

Finally, to explore participants’ most common reasons for their agent preferences, we visually represented key themes and concepts from free-text responses. To do this, we converted text responses into separate word lists for those that explained AI and human preferences, and generated word clouds out of these lists using the wordcloud2 R package (v0.2.2)^104^. Preference ratings below ‘3’ were considered as indicating a preference for AI, ratings of ‘3’ were classified as indicating no preference, and ratings above ‘3’ were interpreted as a preference for the human.

## Data Availability

All materials, data, and analysis scripts are available on the Open Science Framework repository (https://osf.io/wcm75). We have pre-registered our study design and analysis plan, which can be accessed at the same link.
